# ﻿A new Asian lazy toad of the genus *Scutiger* Theobald, 1868 (Anura, Megophryidae) from southern Tibet, China

**DOI:** 10.3897/zookeys.1187.107958

**Published:** 2023-12-20

**Authors:** Sheng-Chao Shi, Lu-Lu Sui, Shun Ma, Fei-Rong Ji, A-Yi Bu-Dian, Jian-Ping Jiang

**Affiliations:** 1 Chengdu Institute of Biology, Chinese Academy of Sciences, Chengdu 610041, China Chengdu Institute of Biology, Chinese Academy of Sciences Chengdu China; 2 University of Chinese Academy of Sciences, Beijing 100049, China University of Chinese Academy of Sciences Beijing China; 3 Mangkang Biodiversity and Ecological Station, Tibet Ecological Safety Monitor Network, Chengdu 854500, China Mangkang Biodiversity and Ecological Station, Tibet Ecological Safety Monitor Network Chengdu China

**Keywords:** Molecular phylogenetic analyses, morphology, *Scutiger*, taxonomy, Tibet Autonomous Region

## Abstract

In this study, a new species named *Scutigerluozhaensis***sp. nov.** is described from Luozha, southern Tibet, China. Genetic analysis based on two mitochondrial genes 16S rRNA and COI and the nuclear gene RAG1 revealed that the new species belongs to an independent phylogenetic clade close to *S.gongshanensis* and *S.nyingchiensis* and shares no RAG1 haplotype with other species. Morphological comparisons based on examined specimens and literatures indicated that it can be diagnosed from congeners by the following combination of characters: (1) body moderate, male body length 47.0–67.2 mm (*n* = 13), female body length 49.8–66.2 mm (*n* = 8); (2) maxillary teeth and budding absent; (3) numerous tiny dense nuptial spines present on dorsal surface of fingers I, II and inner surface of finger III of males in breeding condition with similar size; (4) spine patches on belly of males in breeding condition absent; (5) spines on inner surface of forearm and upper arm of males in breeding condition absent; (6) small patches of black spines present near armpit of males in breeding condition absent; (7) adult males without vocal sac; (8) some large warts and tubercles on dorsum gathered into short skin ridges with several spines present on top; (9) space between upper eyelids wider than upper eyelids; (10) spots or irregular cross bands on limbs absent; (11) webbing between toes rudimentary; (12) coloration of dorsal body olive brown to bronze.

## ﻿Introduction

The Asian lazy toads *Scutiger* Theobald, 1868, is a group of amphibians inhabiting southwestern China, northern Myanmar, Nepal, northern India, and northern Pakistan at altitudes ranging from 1000 to 5300 m (Fei et al. 2009; Fei et al. 2012; Jiang et al. 2020; Frost 2023). Currently, there are 27 valid species in the genus, of which 23 species are distributed in China (Fei et al. 2009; Yang and Huang 2019; Jiang et al. 2020; AmphibiaChina 2023; Frost 2023; Zhou et al. 2023). The species in the genus can be classified into five major clades based on phylogenetic analysis (Hofmann et al. 2017; Yang and Huang 2019; Che et al. 2020; Xing et al. 2023; Zhou et al. 2023):

- Clade A (Medog of eastern Himalaya) including *S.wuguanfui* Jiang, Rao, Yuan, Wang, Li, Hou, Che & Che, 2012 from Medog, southeastern Tibet, China;
- Clade B (central Himalayan clade) including *S.nepalensis* Dubois, 1974 and *S.sikimmensis* (Blyth, 1854) from central Himalaya;
- Clade C (Tsinling Mountains-Sichuan Basin clade) including *S.chintingensis* Liu & Hu, 1960, *S.ningshanensis* Fang, 1985, and *S.feiliangi* Zhou, Guan & Shi, 2023;
- Clade D (from eastern Himalaya and Gaoligong Moutains) including species *S.nyingchiensis* Fei, 1977, *S.spinosus* Jiang, Wang, Li & Che, 2016, *S.tengchongensis* Yang & Huang, 2019, and *S.gongshanensis* Yang & Su, 1979;
- Clade E (Tibet & Hengduan Shan region clade) including eight species *S.boulengeri* (Bedriaga, 1898), *S.mammatus* (Günther, 1896), *S.liupanensis* Huang, 1985, *S.tuberculatus* Liu & Fei, 1979, *S.glandulatus* (Liu, 1950), *S.muliensis* Fei & Ye, 1986, *S.jiulongensis* Fei, Ye & Jiang, 1995, and *S.wanglangensis* Ye & Fei, 2007 from Sichuan, China.

*Scutigerghunsa* Khatiwada, Shu, Subedi, Wang, Ohler, Cannatella, Xie & Jiang, 2019 was weakly supported in the Himalayan clade (Khatiwada et al. 2019) and *S.occidentalis* Dubois, 1978 has an uncertain phylogenetic position from western Himalaya. However, the phylogenetic relationships of the following eight species remain unresolved: *S.adungensi* Dubois, 1979 from eastern Himalaya; *S.bangdaensis* Rao, Hui, Ma & Zhu, 2022 from eastern Tibet; *S.bhutanensis* Delorme & Dubois, 2001 from Bhutan; *S.biluoensis* Rao, Hui, Zhu & Ma, 2022 from Yunnan, China; *S.maculatus* (Liu, 1950) from northwestern Sichuan and eastern Tibet, China; *S.meiliensis* Rao, Hui, Zhu & Ma, 2022 from northwest Yunnan, China; *S.pingwuensis* Liu & Tian, 1978 from Sichuan and southern Gansu, China.

The Paleo-Tibetan region is believed to be the origin of the genus *Scutiger*, and migration across mountains and drainages along the Himalayas is limited (Hofmann et al. 2017). The deep valleys and high mountains of the Himalayas harbor incredible amphibian species diversity, and many have been described recently (e.g., Che et al. 2020). In July 2021 and August 2022, two field surveys were conducted in southern Tibet, and a series of specimens of the genus *Scutiger* were collected from a southern slope of the Himalayas in Luozha County, Shannan Prefecture, Tibet. Subsequent studies on morphological comparisons and molecular analysis reveal that they represent a species new to science, which is described in this study.

## ﻿Materials and methods

### ﻿Sampling

In this study, 34 specimens of *Scutiger* (25 adults, 1 subadult, 2 juveniles, and 6 tadpoles) were collected from Luozha County, Shannan Prefecture, Tibet Autonomous Region, China. The specimens were euthanized and then fixed in 75% ethanol before being deposited in the Herpetology Museum of Chengdu Institute of Biology (**CIB**), Chinese Academy of Sciences. The sex of specimens was determined by the presence of nuptial spines, chest spines, eggs, or examination of gonads when necessary. Tissue samples were taken from legs and preserved separately in 95% ethanol before fixation.

### ﻿Molecular phylogenetic analysis

Total genomic DNA was extracted using QIAamp DNA Mini Kit (QIAGEN, Hilden, Germany) following protocol. Fragments of two mitochondrial genes (16S rRNA and COI) and one nuclear gene (RAG1) were amplified and sequenced. The primer sequences for these genes were retrieved from the literature for 16S rRNA (Simon et al. 1994), COI (Che et al. 2012), and RAG1 (Mauro et al. 2004). PCR amplifications for the gene were performed in a 25 μl volume reaction with the following conditions: an initial denaturing step at 95 °C for 4 min; 36 cycles of denaturing at 95 °C for 40 s, annealing at 52 °C (for COI and RAG1) or 54 °C (for 16S rRNA) for 40 s and extending at 72 °C for 40 s, and a final extending step of 72 °C for 10 min. PCR products were sequenced by Beijing Qingke New Industry Biotechnology Co., Ltd., Beijing, China. Sequences were assembled and aligned using BioEdit v. 7.2.5 (Hall 1999) with default settings and were further revised manually when necessary. The COI and RAG1 sequences were translated to amino acid sequences in MEGA X (Kumar et al. 2018), adjusted for open reading frames, and checked to ensure the absence of premature stop codons. All new sequences were deposited in GenBank.

For phylogenetic analysis, corresponding available sequences of *Scutiger* and three outgroups including *Oreolalaxomeimontis*, *Leptobrachiumboringii*, and *Leptobrachellaliui* were obtained from GenBank in accordance with previous studies (Hofmann et al. 2017; Che et al. 2020). A mitochondrial DNA sequence (16S + COI) matrix was generated for the phylogenetic analyses. Phylogenetic analyses were conducted using maximum likelihood (ML) and Bayesian Inference (BI) methods implemented in Phylosuite (Zhang et al. 2020). Each gene was considered as a partition, and the best evolutionary model was chosen for each partition using PartitionFinder2 (Lanfear et al. 2017) based on Bayesian Inference Criteria (BIC). GTR+G and HKY+G were chosen as the best model for the combined mitochondrial DNA sequences and RAG1 respectively. ML phylogenies were inferred using IQ-TREE (Nguyen et al. 2015) under Edge-linked partition model for 10000 ultrafast bootstraps (Minh et al. 2013), as well as the Shimodaira-Hasegawa-like approximate likelihood-ratio test (Guindon et al. 2010). BI phylogenies were inferred using MrBayes 3.2.6 (Ronquist et al. 2012) under partition model (2 parallel runs, 10 million generations), in which the initial 25% of sampled data were discarded as burn-in. Genetic distances between species for each gene were estimated using MEGA X. All sequences used in this study are listed in Table 1.

**Table 1.** Samples and DNA sequences used in this study.

No.	Species	Locality	Voucher no.	GenBank accession No.			Source
				COI	16S	RAG1	
1	*Scutigerluozhaensis* sp. nov.	Luozha, Tibet, China	CIB QZ2021119	OR141828	OR469879	OR546339	This study
2	*S.luozhaensis* sp. nov.	Luozha, Tibet, China	CIB QZ2021141	OR141835	OR469884	OR546344	This study
3	*S.luozhaensis* sp. nov.	Luozha, Tibet, China	CIB QZ2021139	OR141833	OR469882	OR546342	This study
4	*S.luozhaensis* sp. nov.	Luozha, Tibet, China	CIB 119119	OR141823	OR469858	OR546324	This study
5	*S.luozhaensis* sp. nov.	Luozha, Tibet, China	CIB 119116	OR141831	OR469855	/	This study
6	*S.luozhaensis* sp. nov.	Luozha, Tibet, China	CIB QZ2021117	OR141827	OR469878	OR546338	This study
7	*S.luozhaensis* sp. nov.	Luozha, Tibet, China	CIB QZ2021115	OR141825	/	OR546336	This study
8	*S.luozhaensis* sp. nov.	Luozha, Tibet, China	CIB CJA 20220066	OR141837	OR469864	/	This study
9	*S.luozhaensis* sp. nov.	Luozha, Tibet, China	CIB QZ2021090	OR141824	OR469875	OR546335	This study
10	*S.luozhaensis* sp. nov.	Luozha, Tibet, China	CIB 119117	OR141852	OR469856	OR546322	This study
11	*S.luozhaensis* sp. nov.	Luozha, Tibet, China	CIB 119118	OR141853	OR469857	OR546323	This study
12	*S.luozhaensis* sp. nov.	Luozha, Tibet, China	CIB CJA 20220103	OR141841	OR469868	OR546328	This study
13	*S.luozhaensis* sp. nov.	Luozha, Tibet, China	CIB QZ2021116	OR141826	OR469877	OR546337	This study
14	*S.luozhaensis* sp. nov.	Luozha, Tibet, China	CIB 119120	OR141836	OR469859	OR546325	This study
15	*S.luozhaensis* sp. nov.	Luozha, Tibet, China	CIB 119630-2	OR141843	OR469863	OR546327	This study
16	*S.luozhaensis* sp. nov.	Luozha, Tibet, China	CIB CJA 20220121	OR141849	OR469873	/	This study
17	*S.luozhaensis* sp. nov.	Luozha, Tibet, China	CIB CJA 20220124	OR141851	OR469874	OR546334	This study
18	*S.luozhaensis* sp. nov.	Luozha, Tibet, China	CIB 119630-1	OR141842	OR469862	/	This study
19	*S.luozhaensis* sp. nov.	Luozha, Tibet, China	CIB QZ2021135	OR141830	OR469881	OR546341	This study
20	*S.luozhaensis* sp. nov.	Luozha, Tibet, China	CIB CJA 20220119	OR141847	OR469872	OR546332	This study
21	*S.luozhaensis* sp. nov.	Luozha, Tibet, China	CIB QZ2021140	OR141834	OR469883	OR546343	This study
22	*S.luozhaensis* sp. nov.	Luozha, Tibet, China	CIB QZ2021134	OR141829	OR469880	OR546340	This study
23	*S.luozhaensis* sp. nov.	Luozha, Tibet, China	CIB CJA 20220117	OR141845	OR469870	OR546330	This study
24	*S.luozhaensis* sp. nov.	Luozha, Tibet, China	CIB 119115	OR141832	OR469854	OR546320	This study
25	*S.luozhaensis* sp. nov.	Luozha, Tibet, China	CIB 119122	OR141848	OR469860	/	This study
26	*S.luozhaensis* sp. nov.	Luozha, Tibet, China	CIB 119123	OR141850	OR469861	OR546326	This study
27	*S.luozhaensis* sp. nov.	Luozha, Tibet, China	CIB CJA 20220116	OR141844	OR469869	OR546329	This study
28	*S.luozhaensis* sp. nov.	Luozha, Tibet, China	CIB CJA 20220118	OR141846	OR469871	OR546331	This study
29	*S.luozhaensis* sp. nov.	Luozha, Tibet, China	CIB CJA 20220074	OR141839	OR469866	/	This study
30	*S.luozhaensis* sp. nov.	Luozha, Tibet, China	CIB CJA 20220073	OR141838	OR469865	/	This study
31	*S.luozhaensis* sp. nov.	Luozha, Tibet, China	CIB CJA 20220075	OR141840	OR469867	/	This study
32	*S.gongshanensis*	Gongshan, Yunnan, China	CIB20070717001	KU243062	/	/	Jiang et al. (2016)
33	*S.gongshanensis*	Gongshan, Yunnan, China	CIB20070717002	KU243063	/	/	Jiang et al. (2016)
34	*S.gongshanensis*	—	KIZ020492	/	/	MW111380	Xu et al. (2021)
35	*S.gongshanensis*	—	CAS 234295	/	/	KX208788	Feng et al. (2017)
36	*S.nyingchiensis*	Nyingchi, Tibet, China	KIZ017460	KU243057	/	/	Jiang et al. (2016)
37	*S.nyingchiensis*	Nyingchi, Tibet, China	KIZ017459	KU243056	/	MW111377	Jiang et al. (2016); Xu et al. (2021)
38	*S.nyingchiensis*	Nyingchi, Tibet, China	CAS_XM1095	KY310877	KY310768	/	Hofmann et al. (2017)
39	*S.tengchongensis*	Tengchong, Yunnan, China	SYS a005799	MK121783	MK121789	/	Yang and Huang (2019)
40	*S.spinosus*	Medog, Tibet, China	KIZ011100	KU243054	/	/	Jiang et al. (2016)
41	*S.spinosus*	Medog, Tibet, China	KIZ012645	KU243055	/	/	Jiang et al. (2016)
42	*S.feiliangi*	Luoyang, Henan, China	SYAUBAA000040	OR263444	/	/	Zhou et al. (2023)
43	*S.feiliangi*	Luoyang, Henan, China	SYAUBAA000041	OR263445	/	/	Zhou et al. (2023)
44	*S.ningshanensis*	Shaanxi, China	-	KX619450	KX619450	/	Song et al. (2017)
45	*S.chintingensis*	Tianquan, Sichuan, China	LC141	KY310878	KY310769	KY311042	Hofmann et al. (2017)
46	S.cf.boulengeri	Kangding, Sichuan, China	CIB GGS-MGC4-1	MZ342925	/	/	This study
47	S.cf.boulengeri	Ganzi, Sichuan, China	KQ3_2014	KY310861	KY310751	KY311027	Hofmann et al. (2017)
48	S.cf.boulengeri	Ganzi, Sichuan, China	KQ4_2014	KY310862	KY310752	KY311028	Hofmann et al. (2017)
49	*S.jiulongensis*	Ganzi, Sichuan, China	KIZ045055	KU243066	/	/	Jiang et al. (2016)
50	S.cf.boulengeri	Jone, Gansu, China	jone1	KJ082073	/	/	Hofmann et al. (2017)
51	*S.wanglangensis*	Mianyang, Sichuan, China	21514N1	OQ361635	/	/	Xing et al. (2023)
52	*S.liupanensis*	Jingyuan, Ningxia, China	KIZ NX080514	JN700835	/	/	Che et al. (2012)
53	*S.liupanensis*	/	/	KX352261	KX352261	/	Direct submission
54	*S.liupanensis*	/	KIZNX080519	/	/	MW111376	Xu et al. (2021)
55	*S.mammatus*	Kangding, Sichuan, China	CIB GGS-SDX1-1	MZ342926	MZ351374	/	This study
56	*S.mammatus*	Kangding, Sichuan, China	CIB GGS-PBX4-4	ON422290	ON426806	/	This study
57	*S.mammatus*	Kangding, Sichuan, China	CIB GGS-PBX4-3	ON422291	ON426807	/	This study
58	*S.glandulatus*	Ganzi, Sichuan, China	SC1_2014	KY310879	KY310770	KY311044	Hofmann et al. (2017)
59	*S.glandulatus*	Kangding, Sichuan, China	SH150531	KY310882	KY310773	KY311048	Hofmann et al. (2017)
60	*S.glandulatus*	Ganzi, Sichuan, China	SC2_2014	KY310880	KY310771	KY311045	Hofmann et al. (2017)
61	*S.muliensis*	Yanyuan, Sichuan, China	/	MW167047	EF397277	EF397302	Che et al. (2020); Fu et al. (2007)
62	*S.tuberculatus*	Sichuan, China	/	MW021351	EF397278	EF397299	Che et al. (2020); Fu et al. (2007)
63	*S.boulengeri*	Tageija, Tibet, China	A1-AL	KY310870	KY310760	KY311036	Hofmann et al. (2017)
64	*S.boulengeri*	Muktinath, Mustang district, Nepal	JRK2016-215	MK970610	MK950904	/	Khatiwada et al. (2019)
65	*S.boulengeri*	Damxung, Tibet, China	A3-AL	KY310872	KY310762	KY311038	Hofmann et al. (2017)
66	*S.boulengeri*	Zhaduo, Qinghai, China	JRK2018-03	MK970611	MK950905	/	Khatiwada et al. (2019)
67	*S.boulengeri*	Lhasa, Tibet, China	JS1507_C1	KY310875	KY310765	KY311040	Hofmann et al. (2017)
68	S.cf.mammatus	Gongshan, Yunnan, China	Yako01	/	EU180890	/	Rao and Wilkinson (2007)
69	*S.* sp.	Fugong, Yunnan, China	CAS228188	/	EU180889	/	Rao and Wilkinson (2007)
70	*S.ghunsa*	Ghunsa, Taplejung district, Nepal	JRK2015-193	MK970591	MK950885	/	Khatiwada et al. (2019)
71	*S.occidentalis*	Deosai Plateau, Pakistan	MS_PK6	KY310901	KY310796	KY311066	Hofmann et al. (2017)
72	*S.nepalensis*	Chainpur, Nepal	NME_A2018/13	KY310886	KY310777	KY311052	Hofmann et al. (2017)
73	*S.sikimmensis*	Kongma Danda, Nepal	JS140524	KY310902	KY310798	KY311068	Hofmann et al. (2017)
74	*S.wuguanfui*	Medog, Tibet, China	KIZ011101	KU243060	/	/	Jiang et al. (2016)
75	*S.wuguanfui*	Medog, Tibet, China	KIZ011102	KU243061	/	MW111378	Jiang et al. (2016)
76	*Leptobrachiumboringii*	Mt. Emei, Sichuan, China	Tissue ID: YPX37539	KX812164	KX811930	KX812282	Chen et al. (2017)
77	*Oreolalaxomeimontis*	Mt. Emei, Sichuan, China	CIBEMS18061205	OP247647	MN688660	/	Hou et al. (2020)
78	*Leptobrachellaliui*	Mt. Jinggang, Jiangxi, China	SYSa004045	MH406370	MH406907	MH405153	Liu et al. (2018)

### ﻿Morphological analysis

For adults, measurements were taken with a dial caliper to the nearest 0.1 mm. In total, 24 measurements of 21 adults were measured: **SVL** (snout-vent length: distance from the tip of the snout to the posterior edge of the vent), **AG** (trunk length between axilla and groin: distance between middle point of the two axillae and middle point of groins); **HL** (head length: distance from the rear of the mandible to the tip of the snout); **HW** (head width: distance between the posterior angles of jaw); **HH** (head height: head height at the corner of jaws); **SL** (snout length: distance from tip of snout to anterior border of the orbit); **IND** (internasal distance: distance between inner edge of two nostrils); **IOS** (interorbital space: shortest distance between inner edge of upper eyelids); **UEW** (maximum upper eyelid width); **ACED** (distance between anterior corner of eyes); **PCED** (distance between posterior corner of eyes); **ED** (horizontal eye diameter); **SND** (nostril-snout distance: distance from center of the nostril to tip of the snout); **END** (eye-nostril distance: distance from front of eye to the center of nostril); **LAL** (lower arm length: distance from elbow to wrist); **LAD** (lower arm width: largest diameter of forearm); **HAL** (hand length: distance from wrist to tip of third digit); **HLL** (hindlimb length: distance between vent and tip of fourth toe when leg straightened at right angle to the body); **THL** (thigh length: distance from cloaca to knee); **TL** (tibia length: distance from knee to ankle); **TFL** (tarsal-foot length: length from heel to the tip of the fourth digit); **FL** (foot length: distance from the proximal end of the inner metatarsal tubercle to the tip of the fourth digit); **TW** (tibia width: largest tibia width); **IMTL** (inner metatarsal tubercle length). Morphological terminologies were mostly based on Fei et al. (2009) and webbing formulae are described based on Savage and Heyer (1997). Morphological comparison between the unknown species from Luozha and valid species of genus *Scutiger* were conducted based on data obtained from references (Table 2) and 31 examined specimens of 8 species. For phylogenetical close species, further morphometrics comparison using one-way ANOVA was conducted based on SVL and ratios of another 18 characters to SVL of examined male specimens.

**Table 2.** Morphological comparison of *Scutiger* species based on ten selected characters. Bold typeface indicates characters different from the new species.

Species	Male SVL	Female SVL	Maxillary teeth or budding	Numbers of fingers with nuptial pads	Spine patches pairs on chest	Size of pectoral spine patches vs axillary spine patches	Spine patches on belly in males	Toe webbing	Tubercles on dorsum with spines	Vocal sac	References
*Scutigerluozhaensis* sp. nov.	47.0–67.2 (*n* = 13)	49.8–66.2 (*n* = 8)	absent	I, II, III	2	slightly larger	absent	rudimentary	yes	absent	This study
S.adungensi*	**71–73 (*n* = 2)**	/	**budding**	**I, II**	**1**	/	absent	rudimentary	/	**present**	Dubois (1979)
S.bangdaensis*	45–50 (n=2)	48–50 (n=2)	/	I, II, III	2	larger	absent	**developed**	**no**	/	Rao (2022”2020”)
S.bhutanensis*	53.0–64.9 (*n* = 3)	/	absent	**I, II**	2	similar	absent	rudimentary	/	absent	Delorme and Dubois (2001); Khatiwada et al. (2019)
S.biluoensis*	**73 (*n* = 1)**	53.5 (subadult, *n* = 1)	**teeth**	**I, II**	2	/	absent	rudimentary	/	/	Rao (2022”2020”)
*S.boulengeri*	44.9–53.7 (*n* = 20)	40.2–58.2 (*n* = 8)	absent or only short budding	I, II, III	2	similar	**present**	**well-developed**	yes	absent	Fei et al. (2009); Fei et al. (2012)
*S.chintingensis*	42.0–50.3 (*n* = 22)	48.0–52.8 (*n* = 6)	**teeth**	I, II, III	2	slightly larger	absent	weak	yes	absent	Fei et al. (2009); Fei et al. (2012)
*S.feiliangi*	45.7–50.2 (*n* = 6)	48.9–51.5 (*n* = 3)	**budding**	I, II, III	2	slightly larger	absent	rudimentary	yes	absent	Zhou et al. (2023)
*S.ghunsa*	**42.0–47.8 (*n* = 5)**	50.2–53.9 (*n* = 3)	absent	I, II, III	2	**twice or even larger**	absent	rudimentary	yes	absent	Khatiwada et al. (2019); This study
*S.glandulatus*	**68.0–90.0 (*n* = 17)**	58.0–83.7 (*n* = 14)	absent	**I, II**	2	**twice or even larger**	absent	**developed**	**no**	absent	Fei et al. (2009); Fei et al. (2012)
*S.gongshanensis*	47.0–57.0 (*n* = 21)	49.0–60.0 (*n* = 2)	**budding**	**I, II**	**1**	/	absent	rudimentary	**no**	**present**	Fei et al. (2009); Fei et al. (2012); This study)
*S.jiulongensis*	**67.4–81.5 (*n* = 20)**	/	absent	**I, II**	2	**twice or even larger**	absent	weak	**no**	absent	Fei et al. (2009); Fei et al. (2012)
*S.liupanensis*	40.6–48.0 (*n* = 20)	52.0–59.5 (*n* = 2)	**budding**	I, II, III	2	similar	**present**	rudimentary	yes	absent	Fei et al. (2009); Fei et al. (2012)
S.maculatus*	65.4 (*n* = 1)	69.0 (*n* = 1)	**budding**	I, II, III	2	slightly larger	absent	**developed**	yes	absent	Fei et al. (2009); Fei et al. (2012)
*S.mammatus*	62.4–80.6 (*n* = 20)	60.9–77.8 (*n* = 15)	mostly absent, or with budding	**I, II**	**1**	/	absent	**well developed**	**no**	absent	Fei et al. (2009); Fei et al. (2012)
S.meiliensis*	**70 (*n* = 1)**	65 (*n* = 1)	**teeth**	**I, II**	2	/	absent	rudimentary	/	/	Rao (2022”2020”)
*S.muliensis*	**68.2–80.0 (*n* = 11)**	60.1–67.5 (*n* = 10)	absent	**I, II**	**1**	/	absent	weak	**no**	absent	Fei et al. (2009); Fei et al. (2012); This study)
*S.nepalensis*	**68.0–76.0 (*n* = 8)**	59.5–66.8 (*n* = 4)	/	I, II, III	2	similar	absent	rudimentary	/	absent	Dubois (1974); Khatiwada et al. 2019)
*S.ningshanensis*	51.0 (*n* = 1)	41.0 (*n* = 1)	**teeth**	I, II, III	2	similar	**present**	rudimentary	yes	absent	Fei et al. (2009); Fei et al. (2012)
*S.nyingchiensis*	50.9–67.6 (*n* = 38)	69.6–70.0 (*n* = 3)	**budding**	I, II, III	2	slightly larger	absent	**1/5 webbing on toe IV**	yes	absent	Fei et al. (2009); Jiang et al. (2016); Che et al. (2020); This study
*S.occidentalis*	51–64 (*n* = ?)	/	teeth absent	I, II, III	2	slightly larger	absent	clear (weak via fig. S3.3 of Hofmann et al. 2017)	yes	/	Dubois (1978); Hofmann et al. (2017)
S.pingwuensis*	60.7–75.8 (*n* = 20)	**77.5 (*n* = 1)**	absent	I, II, III	2	**twice or even larger**	**present**	rudimentary	yes	absent	Fei et al. (2009); Jiang et al. (2012)
*S.sikimmensis*	46.9–55.3 (*n* = 28)	50.8–60.5 (*n* = 7)	**budding**	I, II, III	2	slightly larger	absent	rudimentary	yes	absent	Fei et al. (2009); Fei et al. (2012); Che et al. (2020); Frost (2023)
*S.spinosus*	50.5–55.6 (*n* = 12)	53.8–57.2 (*n* = 4)	absent	I, II, III	2	**twice or even larger**	absent	rudimentary	yes	absent	Jiang et al. (2016)
*S.tengchongensis*	**36.0–40.1 (*n* = 8)**	/	absent	I, II, III	2	slightly larger	absent	rudimentary	yes	absent	Yang and Huang (2019)
*S.tuberculatus*	**68.0–76.0 (*n* = 16)**	63.6–79.0 (*n* = 7)	absent	**I, II**	2	**twice or even larger**	absent	rudimentary	**no**	absent	Fei et al. (2009); Fei et al. (2012)
*S.wanglangensis*	52.7–58.2 (*n* = 6)	64.3 (*n* = 1)	**budding**	I, II, III	2	**twice or even larger**	**present**	**1/5 to 1/3 webbing**	yes	absent	Fei et al. (2009); Fei et al. (2012)
*S.wuguanfui*	**77.5–83.8 (*n* = 6)**	**107.4–116.7 (*n* = 2)**	absent	I, II, III	2	similar	absent	rudimentary	yes	**present**	Jiang et al. (2012); Jiang et al. (2016); This study

* Species without available gene data in molecular analysis.

For tadpoles, the stages were identified following Gosner (1960). Thirteen morphometric characters of two tadpoles were measured: **LTRF** (labia tooth row formulae); **TOL** (total length: distance from tip of snout to tip of tail); **BL** (body length: distance from tip of snout to trunk-tail junction); **BH** (maximum body height); **BW** (maximum body width); **SNL** (snout length: distance from tip of snout to anterior border of the orbit); **SSD** (distance from snout to spiraculum: distance from tip of snout to opening of spiraculum); **ODW** (oral disc width: largest width of oral disc); **IOS** (interocular space: minimum distance of eyes); **TMW** (maximum tail muscle width); **TAL** (tail length: distance between posterior side of opening of cloaca to tip of tail); **TMH** (maximum tail muscle height); **HLL** (hindlimb length). Morphological terminologies were based on Fei et al. (2009).

## ﻿Results

### ﻿Molecular analysis

**Figure 1.**
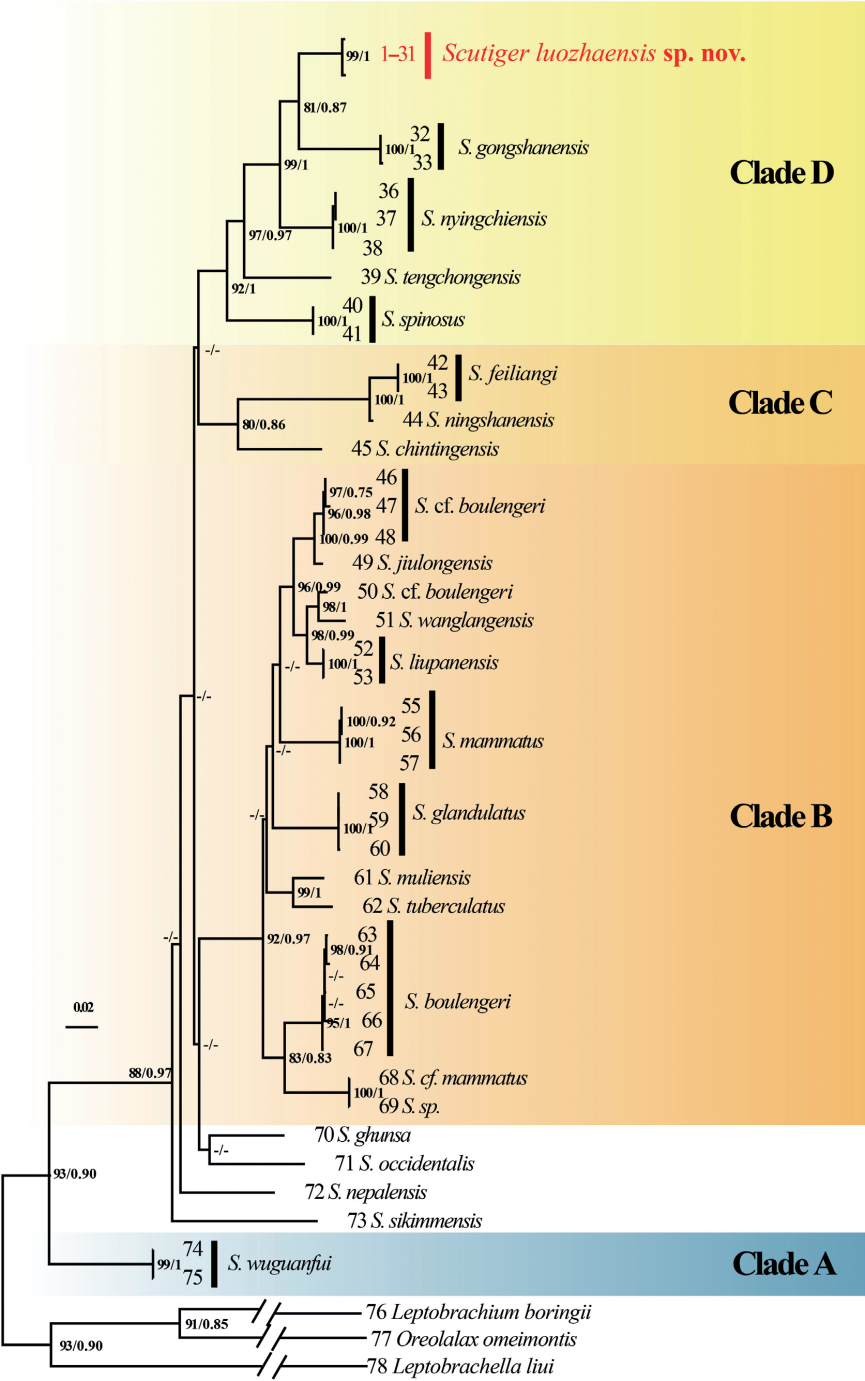
Phylogenetic relationships of *Scutiger* using maximum likelihood (ML) based on the mitochondrial 16S and COI gene sequences. ML bootstrap support/Bayesian posterior probability is denoted beside each node. The symbol “-” represents a value below 70/0.70. For sample numbers refer to Table 1.

**Figure 2.**
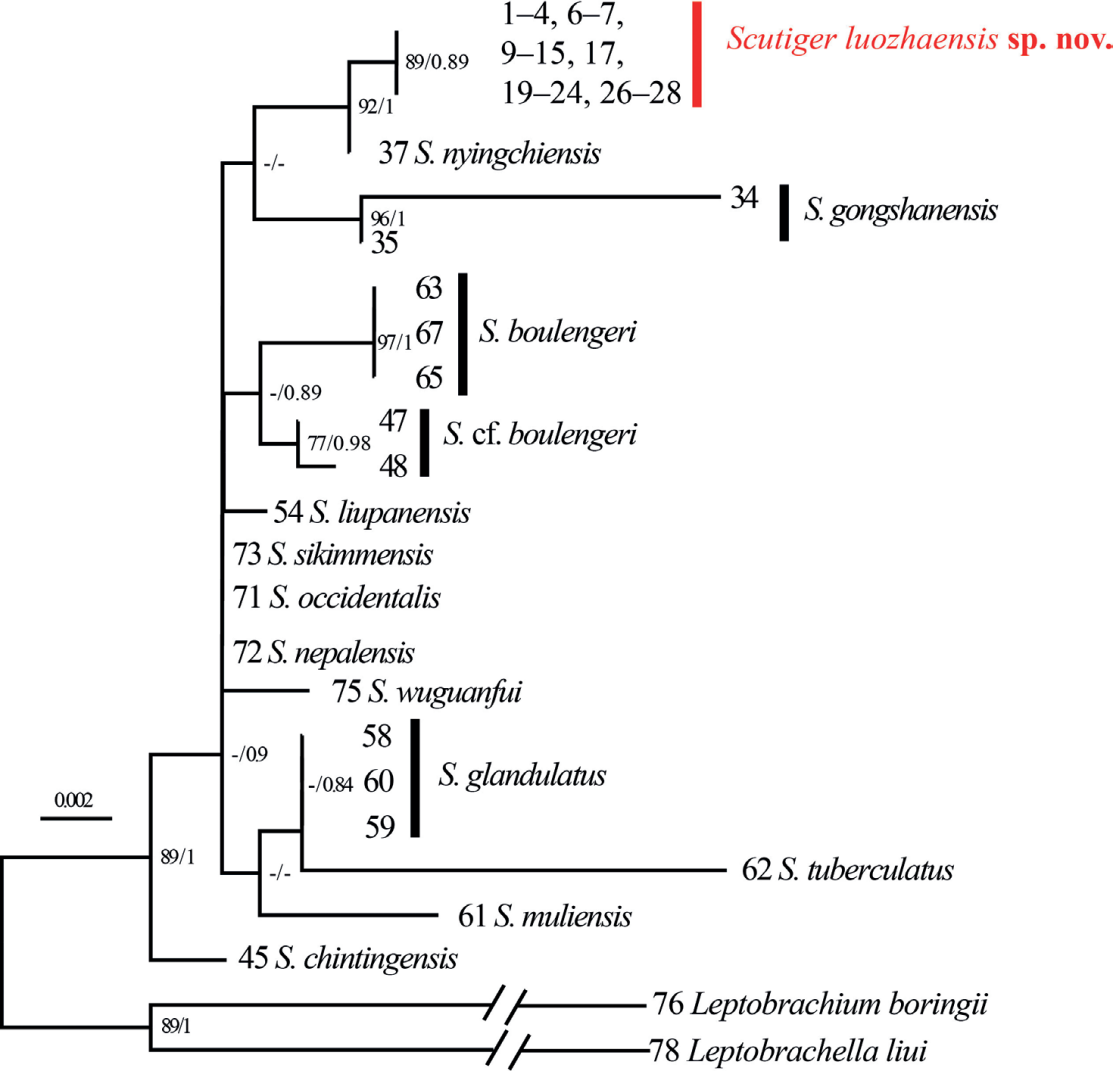
Phylogenetic relationships of *Scutiger* using maximum likelihood (ML) based on RAG1 gene sequences. ML bootstrap support/Bayesian posterior probability is denoted beside each node. The symbol “-” represents a value below 70/0.70. Sample number refer to Table 1.

The aligned sequence matrices of 16S rRNA, COI, and RAG1 genes contain 532 bps, 622 bps, and 1017 bps, respectively. Mitochondrial phylogenetic analysis indicates that *Scutiger* species can be included in five clades, the topology of phylogenetic tree (Fig. 1) is generally similar to those of previous research (Hofmann et al. 2017; Che et al. 2020). Clade A contains only one species, *S.wuguanfui*. Positions for the four species from central and western Himalaya (*S.ghunsa*, *S.occidentalis*, *S.nepalensis*, *S.sikimmensis*) are uncertain. The clade B are the largest, containing *S.boulengeri* species complex, *S.jiulongensis*, *S.wanglangensis*, *S.liupanensis*, *S.mammatus*, *S.glandulatus*, *S.muliensis*, *S.tuberculatus*, and a lineage including S.cf.mammatus and *S.* sp. from Yunnan. The *S.boulengeri* species complex contained three lineages as Lin et al (2023) reported. Clade C contains three species, *S.feiliangi*, *S.ningshanensis* and *S.chintingensis*. The samples collected from Luozha County formed an independent lineage (Luozha lineage) with high bootstrap supports (BS = 99) and Bayesian posterior probabilities (BPP = 1.00). The Luozha lineage further clustered with *S.gongshanensis*, and *S.nyingchiensis* with strong supports (BS = 99, BPP = 1.00). These three species further form clade D with *S.tengchongensis* and *S.spinosus*. Phylogenetic analysis based on RAG1 resulted in a similar topology (Fig. 2). The Luozha lineage is sister to *S.nyingchiensis*.

Genetic distances between species of *Scutiger* are shown in Suppl. materials 1–3 based on 16S rRNA, COI, and RAG1 genes, respectively. The smallest genetic distances between the Luozha lineage and other taxa of *Scutiger* based on 16S rRNA and COI are 0.026–0.030 and 0064–0.068 respectively (vs *S.nyingchiensis*). These are comparable or larger than multiple known species pair (e.g., *S.glandulatus* vs *S.jiulongensis* 0.066–0.068 for COI; *S.liupanensis* vs *S.mammatus* 0.024 for 16S rRNA, 0.064–0.068 for COI; *S.tengchongensis* vs *S.chintingensis* 0.029 for 16S rRNA, *S.muliensis* vs *S.tuberculatus* 0.008 for 16S rRNA, 0.055 for COI). The genetic distances for nuclear gene RAG1 between the Luozha lineage and other species are much smaller (0.001–0.020); however, three species (*S.occidentalis*, *S.nepalensis*, *S.sikimmensis*) even share the same RAG1 haplotype.

### ﻿Morphological results

**Figure 3.**
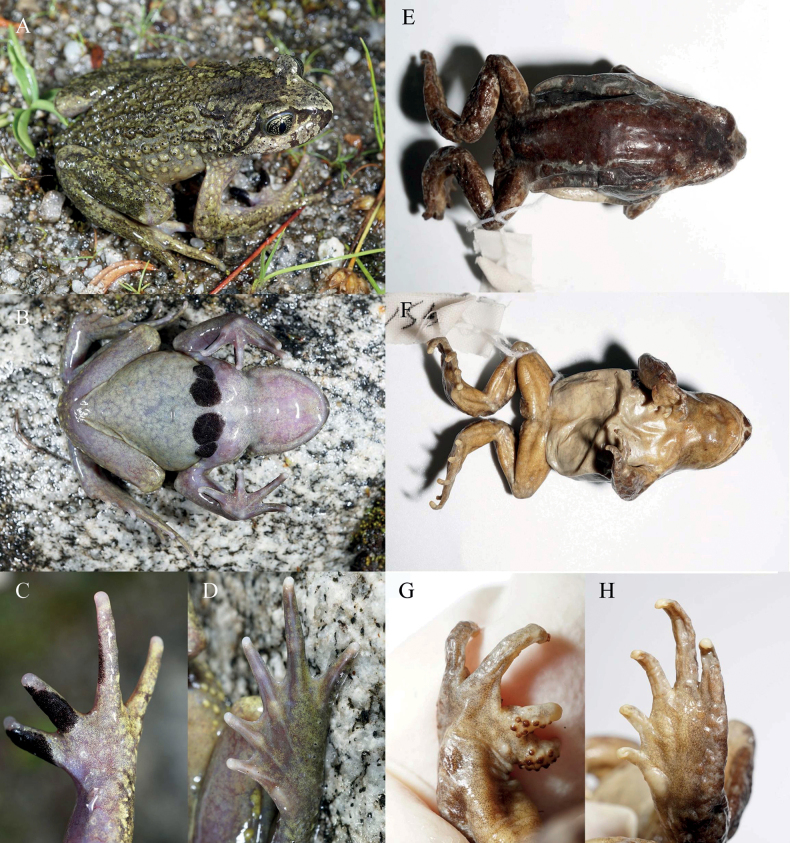
Adult male *Scutigernyingchiensis* (CIB QX207) from Nyingchi, Tibet, China (**A–D**) and adult male *S.gongshanensis* (KIZ 036221) from Gongshan, Yunnan, China (**E–H**) **A** dorsolateral body **B** ventral body **C** dorsal right hand and **D** ventral foot **E** dorsolateral body **F** ventral body **G** dorsal right hand and **H** ventral foot. Photographed by SCS.

Comparisons based on ten selected morphological characters for all *Scutiger* species are summarized in Table 2. Images of two phylogenetically close related species to the Luozha lineage (*S.nyingchiensis* and *S.gongshanensis*) were demonstrated in Fig. 3. The Luozha lineage is morphologically distinguished from other known species of *Scutiger* based on a combination of morphological features as follows:

For *S.adungensi*, the Luozha lineage differs by absence of vocal sac for adult males(vs presence); absence of maxillary teeth and budding (vs presence of budding); smaller male body size (SVL 47.0–67.2 mm vs 71–73 mm); presence numerous dense tiny black nuptial spines present on dorsal surface of fingers I, II and inner surface of finger III of males in breeding condition (vs large spines on inner two fingers); two pair of spine patches on chest of breeding male (vs one pair).

For *S.bangdaensis*, the Luozha lineage differs by presence of one to six separated spines on top of each dorsal tubercle of males in breeding condition (vs absence of spines on tubercles); rudimentary webbing between toes (vs developed).

For *S.bhutanensis*, the Luozha lineage differs by numerous dense tiny black nuptial spines present on dorsal surface of fingers I, II and inner surface of finger III of males in breeding condition (vs 16–18 large nuptial spines on each of inner two fingers of males); space between upper eyelids being wider than upper eyelids (vs narrower); forearm being longer than hand (vs equal); relatively larger feet in males (FL/SVL 40.9–50.4% vs 38.5%).

For *S.biluoensis*, the Luozha lineage differs by absence of maxillary teeth (vs presence); presence of nuptial spines on dorsal surface of fingers I, II and inner surface of finger III of males in breeding condition (vs on inner two fingers); smaller male body size (SVL 47.0–67.2 mm vs 73 mm).

For *S.boulengeri*, the Luozha lineage differs by absence of spine patches on belly of males in breeding condition (vs presence); rudimentary webbing between toes (vs well-developed webbing); coloration of dorsal body olive brown to bronze (vs greyish olive).

For *S.chintingensis*, the Luozha lineage differs by absence of spines on inner surface of upper arm and forearm of males in breeding condition (vs presence); rudimentary webbing between toes (vs 1/3 webbing between toes); absence of maxillary teeth (vs developed maxillary teeth); absence of a pair of long glandular skin ridges on shoulder or middle dorsum (vs presence); absence of femoral glands (vs presence).

For *S.feiliangi*, the Luozha lineage differs by presence of one to six separated spines on top of each dorsal tubercle of males in breeding condition (vs a layer of keratinized dense tiny spines on tubercles on dorsum of both gender in breeding); absence of spines on inner surface of forearm of males in breeding condition (vs presence); upper and lower half of iris uniformly bicolored (vs upper half golden, lower half brown).

For *S.ghunsa*, the Luozha lineage differs by pectoral spine patches being slightly larger than the axillary spine patches (vs twice or even larger); larger male body size (SVL 47.0–67.2 mm vs 42.0–47.8 mm); the yellow tubercles scattered around cloaca of males in breeding condition (vs creamy white granules surrounding vent); coloration of dorsal body olive brown to bronze (vs pale brown); absence of dark brown bands on upper lip (vs present); absence of irregular black spots on limbs (vs present).

For *S.glandulatus*, the Luozha lineage differs by smaller and moderate male body size (SVL 47.0–67.2 mm vs 68.0–90.0 mm, body stout); presence of nuptial spines on dorsal surface of fingers I, II and inner surface of finger III of males in breeding condition (vs on inner two fingers); pectoral spine patches being slightly larger than the axillary spine patches (vs twice or even larger); rudimentary webbing between toes (vs well-developed webbing); presence of spine on warts and tubercles on dorsum (vs absence).

For *S.gongshanensis*, the Luozha lineage differs by absence of vocal sac for adult males (vs presence); absence of maxillary teeth and budding (vs presence of budding); presence of numerous tiny dense nuptial spines on dorsal surface of fingers I, II, and inner surface of finger III of males in breeding condition (vs large spines on inner two fingers); two pair of spine patches on chest of breeding male (vs one pair); presence of spine on warts and tubercles on dorsum (vs absence); absence of a wide dark strip on dorsum from behind eyes to vent (presence) (Fig. 3).

For *S.jiulongensis*, the Luozha lineage differs by smaller and moderate male body size (SVL 47.0–67.2 mm vs 67.4–81.5 mm, body stout); presence of nuptial spines on dorsal surface of fingers I, II and inner surface of finger III of males in breeding condition (vs on inner two fingers); pectoral spine patches being slightly larger than the axillary spine patches (vs twice or even larger); presence of one to six separated spines on top of each dorsal tubercle of males in breeding condition (vs absence of spines on tubercles).

For *S.liupanensis*, the Luozha lineage differs by absence of maxillary teeth and budding (vs presence of budding); absence of spine patches on belly of males in breeding condition (vs presence); absence of a pair of large tubercles around cloaca (vs presence).

For *S.maculatus* the Luozha lineage differs by absence of maxillary teeth and budding (vs presence of budding); rudimentary webbing between toes (vs well-developed webbing).

For *S.mammatus*, the Luozha lineage differs by moderate body (vs stout body); presence of nuptial spines on dorsal surface of fingers I, II and inner surface of finger III of males in breeding condition (vs on inner two fingers); two pair of spine patches on chest of breeding male (vs one pair); rudimentary webbing between toes (vs well-developed webbing); presence of one to six separated spines on top of each dorsal tubercle of males in breeding condition (vs absence of spines on tubercles).

For *S.meiliensis*, the Luozha lineage differs by absence of maxillary teeth and budding (vs presence of teeth); presence of nuptial spines on dorsal surface of fingers I, II and inner surface of finger III of males in breeding condition (vs on inner two fingers); smaller male body size (SVL 47.0–67.2 mm vs 70 mm).

For *S.muliensis*, the Luozha lineage differs by moderate body and smaller male body size (SVL 47.0–67.2 mm vs stout body, 68.2–80.0 mm); presence of nuptial spines on dorsal surface of fingers I, II and inner surface of finger III of males in breeding condition (vs on inner two fingers); two pair of spine patches on chest of breeding male (vs one pair); presence of spine on warts and tubercles on dorsum (vs absence).

For *S.nepalensis*, the Luozha lineage differs by smaller male body size (SVL 47.0–67.2 mm vs 68.0–76.0 mm); head width being smaller than (males) or subequal to (females) tibia length (vs head width equal or greater than tibia length for males and females of *S.nepalensis* respectively).

For *S.ningshanensis*, by absence of maxillary teeth and budding (vs presence of teeth); absence of spine patches on belly of males in breeding condition (vs presence); dozens of yellow tubercles scattered around cloaca of males in breeding condition (vs a pair of white glands around vent); absence of a blue spot on tip of snout (vs present).

For *S.nyingchiensis*, the Luozha lineage differs by rudimentary webbing between toes (webbing formula I½–1II½–2III1½–2½IV2½–2V vs 1/5 webbing on toe IV, webbing formula I0–½II0–1½III1–2IV2–1½V); absence of maxillary teeth and budding (vs presence of budding); coloration of dorsal body olive brown to bronze (vs greyish olive) (Fig. 3).

For *S.occidentalis*, the Luozha lineage differs by coloration of dorsal body olive brown to bronze (vs greyish olive, e.g., fig. S3.2. of Hofmann et al. 2017); rudimentary webbing between toes (vs clear webbing between toes).

For *S.pingwuensis* the Luozha lineage differs by smaller female body size (SVL 49.8–66.2 mm vs 77.5 mm); pectoral spine patches being slightly larger than the axillary spine patches (vs twice or even larger); absence of spine patches on belly of males in breeding condition (vs presence); absence of spines on inner surface of upper arm and forearm of males in breeding condition (vs presence).

For *S.sikimmensis*, the Luozha lineage differs by absence of maxillary teeth and budding (vs presence of budding); presence of numerous tiny dense nuptial spines on dorsal surface of fingers I, II and inner surface of finger III of males in breeding condition (vs large spines on inner two fingers, small spines on inner surface of third finger); absence of distinct irregular cross bands on limbs (vs presence); space between upper eyelids being wider than upper eyelids (vs narrower).

For *S.spinosus*, the Luozha lineage differs by some large warts and tubercles on dorsum gathered into short skin ridges with several spines present on top (vs prominent, conical-shaped tubercles on dorsal and lateral surfaces independent, each tubercle bearing only one black spine); absence of small patches of black spines present near armpit of males in breeding condition (vs presence); pectoral spine patches being slightly larger than the axillary spine patches (vs pectoral twice longer than axillary); absence of cross bands on limbs (vs present).

For *S.tengchongensis*, the Luozha lineage differs by larger male body size (SVL 47.0–67.2 vs 36.0–40.1 mm); absence of small patches of black spines present near armpit of males in breeding condition (vs presence); numerous tiny dense nuptial spines on dorsal surface of fingers I, II and inner surface of finger III of males in breeding condition with similar size (vs black nuptial spines on the first and second fingers being larger than those on the third finger); coloration of dorsal body olive brown to bronze (vs reddish brown).

For *S.tuberculatus*, the Luozha lineage differs by moderate and smaller male body (SVL 47.0–67.2 mm vs stout body 68.0–76.0 mm); numerous tiny dense nuptial spines on dorsal surface of fingers I, II and inner surface of finger III of males in breeding condition (vs large spines on dorsal surface of fingers I, II); pectoral spine patches being slightly larger than the axillary spine patches (vs twice or even larger); presence of spines on tubercles on dorsum (vs absence of spines on large warts on dorsum).

For *S.wanglangensis*, the Luozha lineage differs by absence of maxillary teeth and budding (vs presence of budding); pectoral spine patches being slightly larger than the axillary spine patches (vs twice or even larger); absence of spine patches on belly of males in breeding condition (vs presence); absence of small patches of black spines present near armpit of males in breeding condition (vs presence); rudimentary webbing between toes (vs 1/5 to 1/3 webbing); coloration of dorsal body olive brown to bronze (vs greyish olive); absence of a longitudinal strip on middle dorsum connecting with brown triangle between eyes (vs presence).

For *S.wuguanfui*, the Luozha lineage differs by moderate and smaller body (SVL 47.0–67.2 mm for male, 49.8–66.2 mm for female vs stout body, 77.5–83.8 mm for male, 107.4–116.7 for female); absence of vocal sac for adult males (vs presence of an internal single subgular vocal sac for males); absence of numerous small black spines on upper chest (vs presence); space between upper eyelids being wider than upper eyelids (vs narrower).

Morphometric comparisons based on 19 characters between the Luozha lineage and two phylogenetically close species *S.gongshanensis* and *S.nyingchiensis* are shown in Table 3 (detailed data provided in Suppl. material 4). The Luozha lineage further differs from *S.gongshanensis* in HL/SVL, LAL/SVL, LAW/SVL, HLL/SVL, THL/SVL, TFL/SVL, FL/SVL, and *S.nyingchiensis* in IND/SVL, LAL/SVL, LAW/SVL.

**Table 3.** Morphometric comparisons between Luozha lineage, *S.nyingchiensis*, and *S.gongshanensis*. *P*-values were obtained from the one-way ANOVA for the male group. Significance was set at *P* = 0.05. Bolded numbers indicate significant *P*-values.

Characters	Luozha lineage (A, *n* = 9)				*S.nyingchiensis* (B, *n* = 8)				*S.gongshanensis* (C, *n* = 2)				A vs B	A vs C	B vs C
	Min	Max	Average	SD	Min	Max	Average	SD	Min	Max	Average	SD			
SVL	47	57.9	54	3.5	50.9	55.6	53.9	1.6	51	53.2	52.1	1.1	0.962	0.423	0.445
HL/SVL	28.2%	34.3%	30.5%	1.7%	30.0%	34.4%	32.7%	1.4%	31.0%	32.0%	31.5%	0.5%	**0.018**	0.487	0.377
HW/SVL	30.5%	37.1%	33.8%	2.1%	32.2%	35.2%	33.8%	0.9%	32.2%	34.4%	33.3%	1.1%	0.957	0.707	0.735
SL/SVL	11.4%	14.7%	12.8%	1.0%	12.3%	14.4%	13.4%	0.7%	13.0%	13.5%	13.2%	0.3%	0.186	0.538	0.822
IND/SVL	8.9%	10.3%	9.6%	0.5%	7.9%	10.0%	8.7%	0.6%	8.2%	9.2%	8.7%	0.5%	**0.007**	0.069	0.985
IOS/SVL	8.0%	10.9%	9.0%	1.0%	7.8%	9.6%	8.6%	0.6%	8.6%	8.8%	8.7%	0.1%	0.307	0.628	0.875
UEW/SVL	5.9%	10.3%	7.6%	1.3%	6.5%	7.9%	7.3%	0.4%	7.3%	7.7%	7.5%	0.2%	0.615	0.909	0.844
ACED/SVL	13.5%	18.8%	16.4%	1.7%	14.8%	17.9%	16.3%	1.0%	15.5%	18.0%	16.8%	1.3%	0.867	0.771	0.697
PCED/SVL	23.9%	31.1%	27.3%	2.3%	6.7%	27.1%	21.4%	8.4%	24.9%	27.6%	26.3%	1.4%	0.069	0.832	0.339
ED/SVL	8.1%	11.3%	9.8%	1.0%	9.4%	10.8%	10.1%	0.5%	10.2%	10.4%	10.3%	0.1%	0.471	0.478	0.794
LAL/SVL	25.1%	32.1%	27.7%	2.1%	23.6%	27.6%	25.8%	1.2%	19.4%	20.4%	19.9%	0.5%	**0.040**	**0.000**	**0.040**
LAW/SVL	9.2%	12.7%	10.4%	1.0%	11.0%	14.1%	12.1%	0.9%	11.5%	13.9%	12.7%	1.2%	**0.004**	**0.014**	**0.004**
HAL/SVL	21.9%	27.9%	25.0%	1.9%	23.1%	26.9%	25.3%	1.3%	22.2%	22.7%	22.5%	0.3%	0.728	0.085	0.059
HLL/SVL	136.2%	164.3%	146.0%	9.6%	143.0%	155.6%	149.2%	3.6%	103.0%	104.9%	104.0%	0.9%	0.390	**0.000**	**0.000**
THL/SVL	36.3%	48.9%	41.4%	4.1%	41.1%	44.8%	43.2%	1.1%	33.5%	37.6%	35.6%	2.0%	0.290	**0.036**	**0.010**
TL/SVL	36.3%	43.8%	38.4%	2.5%	38.5%	42.5%	40.0%	1.3%	34.1%	36.7%	35.4%	1.3%	0.126	0.087	**0.013**
TFL/SVL	62.5%	73.6%	66.2%	3.7%	63.4%	69.4%	66.0%	1.9%	52.7%	59.6%	56.2%	3.4%	0.917	**0.001**	**0.002**
FL/SVL	42.1%	50.4%	45.8%	2.6%	42.6%	50.3%	45.6%	2.6%	38.3%	38.8%	38.6%	0.2%	0.849	**0.003**	**0.005**
IMTL/SVL	4.4%	8.3%	6.6%	1.2%	5.3%	8.5%	6.7%	0.9%	5.9%	6.6%	6.2%	0.3%	0.849	0.672	0.593

In conclusion, the unknown *Scutiger* from Luozha presents an independent lineage with interspecific genetic divergence, and it is morphologically distinct from all known species. It is diagnosed as a new species and hence described herein.

### ﻿Taxonomic account

#### ﻿ Scutiger luozhaensis sp. nov.

Taxon classificationAnimaliaAnuraMegophryidae

11764269-F481-5CC0-BD5D-9CABF96DBAB4

https://zoobank.org/4DD29214-7B65-484E-A51F-297C8BF26545

**Figure 4.**
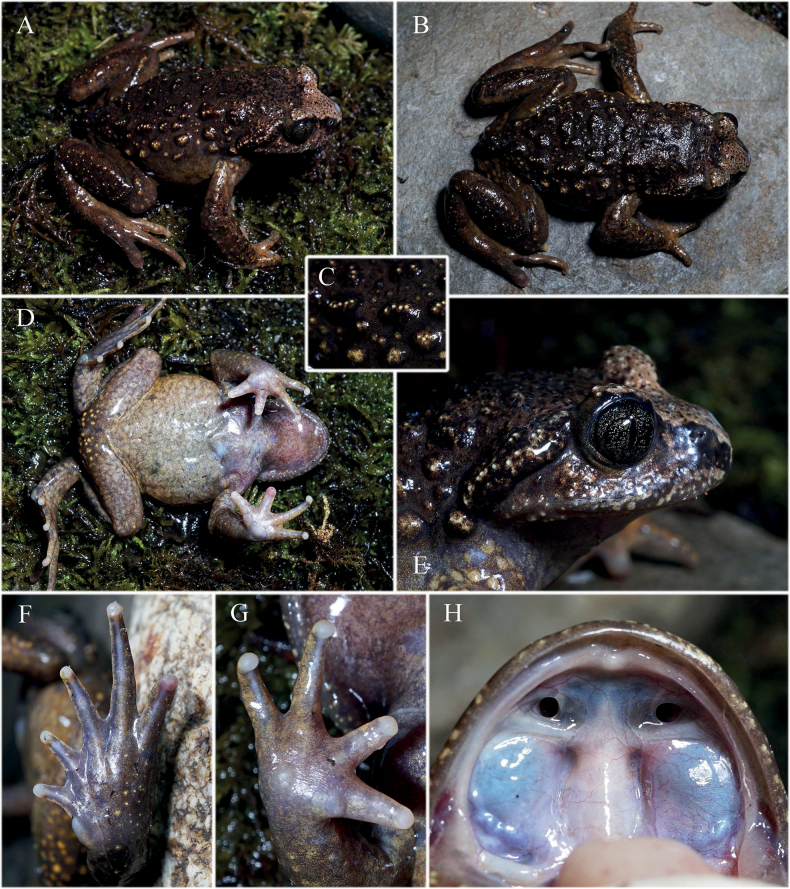
Holotype of *Scutigerluozhaensis* sp. nov. (CIB 119115) in life **A** dorsolateral body **B** dorsal body **C** short skin ridges and tubercles on dorsal body **D** ventral body **E** lateral head **F** ventral feet **G** ventral hand **H** ventral view of maxillary showing no vomerine or maxillary teeth. Photographed by SCS.

**Figure 5.**
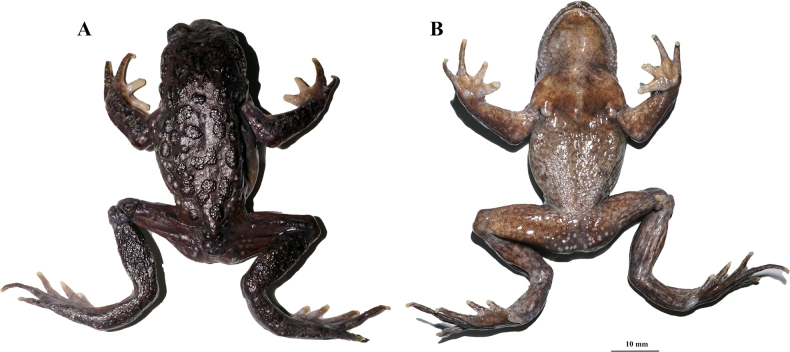
Holotype of *Scutigerluozhaensis* sp. nov. (CIB 119115) in preservative **A** dorsal and **B** ventral body. Photographed by LLS.

**Figure 6.**
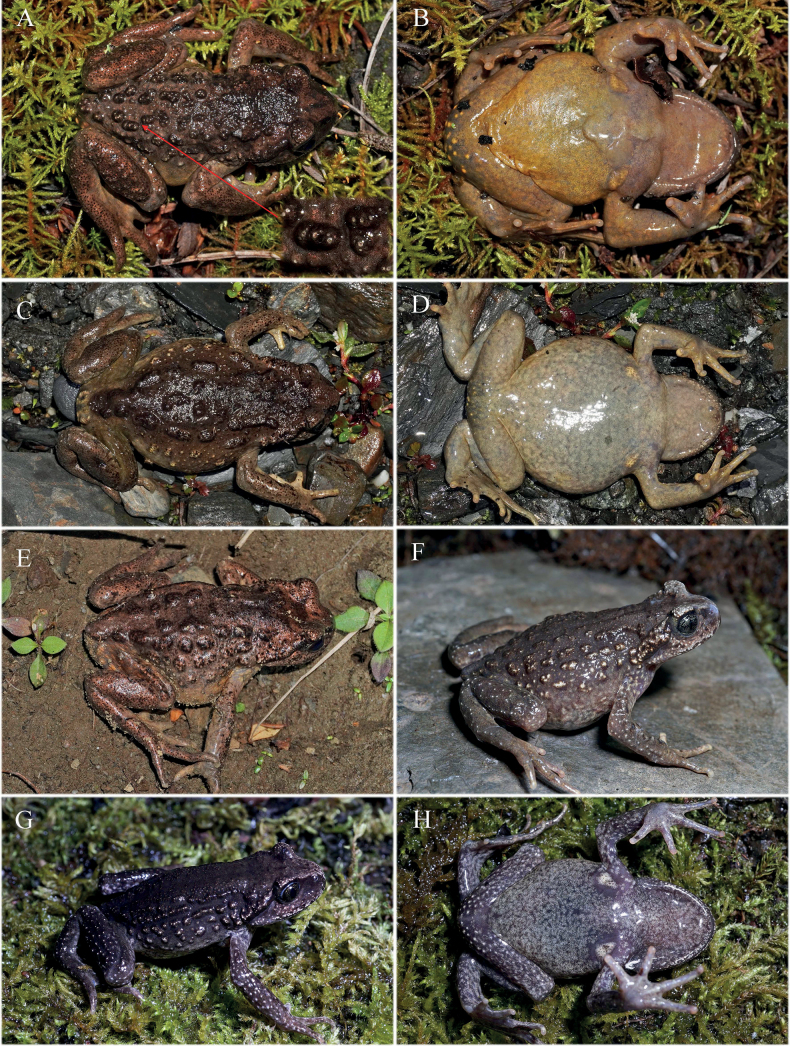
Variations of *Scutigerluozhaensis* sp. nov. **A, B** dorsal and ventral view of an adult male from Lakang Town **C, D** dorsal and ventral view of an adult female from Lakang Town **E** dorsolateral view of an adult female from Lakang Town **F** dorsolateral view of adult female CIB 119120 from Se Town **G, H** dorsolateral and ventral view of juvenile CIB QZ2021115 from Shengge Town. Photographed by SCS.

**Figure 7.**
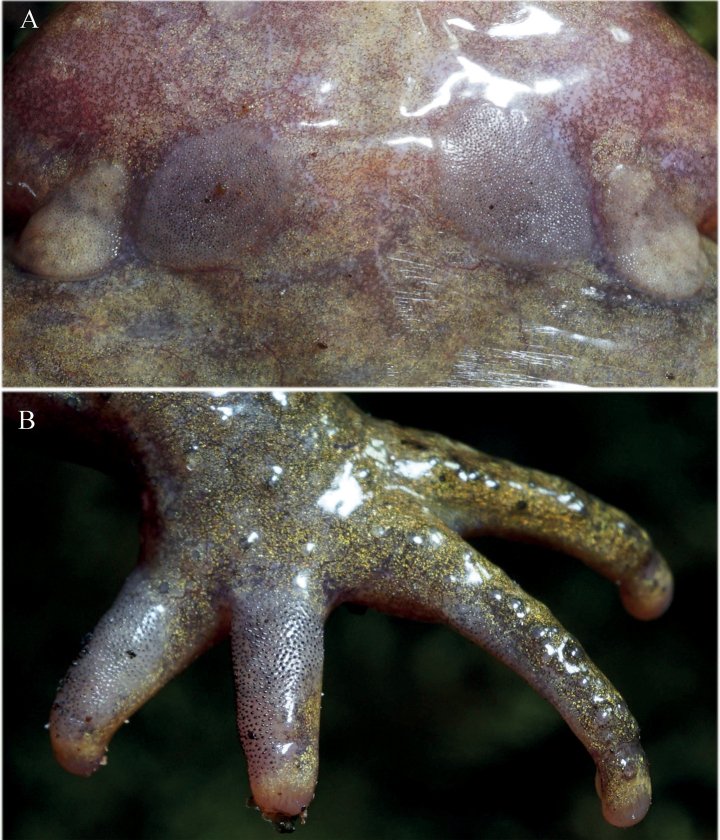
Spine patches and nuptial spines of a male *Scutigerluozhaensis* sp. nov. CIB QZ2021090 from Shengge Town, Luozha County **A** outer smaller axillary spine patches and inner larger pectoral spine patches **B** tiny dense nuptial spines on dorsal surfaces of fingers I, II, and inner surface of finger III. Photographed by SCS.

**Figure 8.**
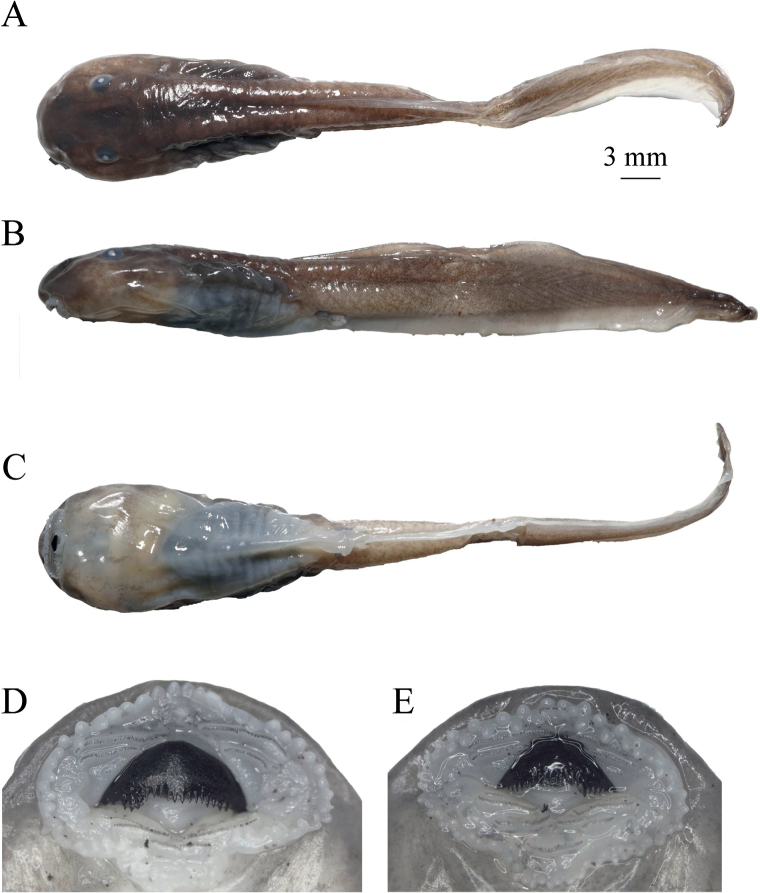
Tadpole of *Scutigerluozhaensis* sp. nov. at Gosner stage 29 from Lakang Town, Luozha County **A** dorsal view **B** lateral view **C** ventral view **D** mouth part of CIB 119630-1 **E** mouth area of CIB 119630-2. Photographed by LLS.

Figs 4 , 5 , 6 , 7 , 8 ; Tables 3 , 4 , 5 ; Suppl. materials 3 , 4

##### Type material.

***Holotype***: CIB 119115, adult male, collected from Gari Village, Se Town, Luozha County, Tibet, China (28.2413°N, 90.7842°E, 4150 m a.s.l.) by Sheng-Chao Shi, Peng Yan and Shun Ma on August 3^rd^, 2021. The holotype was found on alpine meadow aside a stream at night.

***Paratypes***: 8 specimens: CIB 119116, adult male, collected at the same date and location as holotype; CIB 119117–119118, two adult males, and CIB 119122–119123, two adult females collected at Gari village, Se Town (28.2209°N, 90.8290°E, 3970 m a.s.l.); CIB 119119, adult female, collected adjacent to holotype at Gari village, Se Town (28.2216°N, 90.8283°E, 3795 m a.s.l.); CIB 119120, adult female, collected adjacent to holotype at Gari village, Se Town (28.2459°N, 90.7772°E, 4228 m a.s.l.); CIB 119121, adult female, collected at Quzangbu valley, Lakang Town (28.1189°N, 91.1862°E, 3667 m a.s.l.).

##### Diagnosis.

*Scutigerluozhaensis* sp. nov. is assigned to the genus *Scutiger* by the followings: (1) maxillary teeth absent or indistinct; (2) vomerine teeth absent; (3) tympanum and tympanic ring entirely absent; (4) pupil vertical, (5) femoral glands indistinct; (6) pectoral and axillary gland present in males, and covered by black spines in males in breeding condition; (7) inner three fingers with black nuptial spines in males in breeding condition (Fei et al. 2009; Fei and Ye 2016).

*Scutigerluozhaensis* sp. nov. is diagnosed from its congeners by a combination of the following characters: (1) body moderate, male body length 47.0–67.2 mm (*n* = 13), female body length 49.8–66.2 mm (*n* = 8); (2) maxillary teeth and budding absent; (3) numerous tiny dense nuptial spines present on dorsal surface of fingers I, II and inner surface of finger III of males in breeding condition with similar size; (4) spine patches on belly of males in breeding condition absent; (5) spines on inner surface of forearm and upper arm of males in breeding condition absent; (6) small patches of black spines present near armpit of males in breeding condition absent; (7) adult males without vocal sac; (8) some large warts and tubercles on dorsum gathered into short skin ridges with several spines present on top; (9) space between upper eyelids wider than upper eyelids; (10) spots or irregular cross bands on limbs absent; (11) webbing between toes rudimentary; (12) coloration of dorsal body olive brown to bronze.

##### Description of holotype.

Adult male, body moderate (SVL 56.4, body weighted 12.5 g in life, all morphometric measurements in mm).

***Head*** small (HW 17.2, HL 16.6, HH 9.4, ACED 8.0, PCED 13.7), nearly wide as long (HW/HL 1.04), relatedly flat (HH/HW 0.55); snout short (SL 6.5), rounded, slightly protruding beyond jaw, rostral appendage absent, canthus rostralis obtuse, loreal region oblique and concave; nostril oval, closer to tip of snout than eyes (SND 3.3, END 4.1); internarial distance larger than distance from anterior margin of eye to nostril (IND/END 1.22); eyes moderate in size (ED 5.9, ED/HL 0.36); pupil narrow and vertical; distance between upper eyelids smaller than distance between nostrils, but larger than upper eyelids width (IOS 4.5, IND 5.0, UEW 3.8), interorbital space flat; tympanum absent; supratympanic ridge thick, from posterior part of upper eyelids to shoulder; pineal ocellus not present; maxillary teeth and budding absent; tongue oval, not emarginate behind, without papillae and medial lingual sulcus; choanae oval, located against anterior border of palate, widely separated; vomerine ridges and vomerine teeth absent; choana small and oval, widely apart from each other; vocal sac and openings absent.

***Forelimbs*** long (LAL 14.6, LAW 5.6, HAL 12.6, LAW/LAL 0.38); fingers slender, without web and lateral dermal fringes, relative length of fingers: I<II<IV<III; fingertips rounded, not dilated; subarticular tubercles and supernumerary tubercles below the base of finger absent; inner metacarpal tubercles distinct and flat, positioned at the base of finger I, slightly smaller than outer metacarpal tubercles; nuptial pad present on dorsal surface of finger I, II and inner surface of finger III, nuptial spines on finger I and II numerous dense and tiny, but faded on finger III.

***Hindlimbs*** moderately short (TL 21.0, TL/SVL 0.37); tibiotarsal articulation reaching the shoulder when hindlimbs stretching forward; heels widely separated when hind limbs are flexed and held perpendicular to body; thighs slightly longer to tibia but shorter than feet (THL 21.4, TL 21.0, FL 25.0, TFL 36.0); tibia moderate (TW 5.9, TW/THL 0.28); toes slender, relatively lengths I<II<V<III<IV, rudimentary webbed, webbing formula: I½–1II½–2III1½–2½IV2½–2V, with narrow lateral fringes, tips rounded and not dilated; subarticular tubercles indistinct; dermal ridges continuous on under toes; inner metatarsal tubercle elliptical and prominent (IMTL 4.1), outer metatarsal tubercle absent; tarsal fold thick.

***Skins*** rather rough on dorsal surface; large warts and tubercles scattered on dorsal body, some arranged in rows, some gathered into short skin ridges; keratinized spines on warts and tubercles not observed, but there are one to six separated pale colored tiny granules on top of each dorsal tubercle (Fig. 4C), presumedly to be remains of faded keratinized spines; skins on head between and before eyes relatively smooth; tiny pale-colored granules also present along supratympanic ridge and on upper eyelids; temporal region with several small granules, loreal region relatively smooth; upper and lower lips without spines present but also has tiny pale-colored granules, spines presumedly to be faded with the ending of breeding season; skins on dorsal limbs thick, scattered with small granules; skin on dorsal tibiotarsal region with developed dermal glands; dorsolateral skin folds absent; ventral body, flanks and ventral limbs smooth; ventral hands and feet smooth; dozens of fine rounded tubercles scattered around cloaca; a pair of pectoral spine patches faded but with a pair of pectoral skin pads left on chest; axillary glands present and relatively smaller, spines on axillary glands had faded; femoral glands absent.

##### *Coloration*.

In life (Fig. 4), skin on dorsal surface of body and limbs basically deep olive brown; anterior head pale brown; granules on top of warts and tubercles of dorsal body, limbs, supratympanic ridge and upper eyelids pale yellowish; tubercles around cloaca yellow; ventral surface generally olive grey, more purplish on chest and throat; ventral hands and feet olive yellow; nuptial spines on fingers black; iris basically dark, with numerous bronze pigments and irregular dark gaps; tongue flesh colored. In preservative (Fig. 5), dorsal body mostly black-brown, tubercles on flanks nearly black with yellowish white point, dorsal surface of finger I, II and inner surface of finger III pale yellowish; ventral belly pale grayish brown; ventral limbs, ventral chest and ventral head yellowish brown; granules on lips, metacarpal tubercles, tubercles around cloaca and axillary glands grey; iris dark with uniformly distributed bronze pigments, upper and lower half not bicolored.

##### Variation

Morphological measurements of the adult type series are summarized in Table 4 (see detailed measurements in Suppl. material 5). The other specimens generally exhibit morphological consistency with the holotype, albeit with some variations. The arrangement and shape of large spiny tubercles on the dorsal surface of body vary among individuals, but all have large longitudinal tubercles; warts and tubercles on dorsum are larger and fewer on some females (Fig. 6C, D), and smaller on juvenile (Fig. 6G). Dorsal coloration varies from olive brown to bronze in adults (Fig. 6A, C, E, F), while darker in some juveniles (Fig. 6G, H). Ventral coloration varies from olive grey to immaculate yellowish (Fig. 6B). Keratinized spines on warts and tubercles did not fade on a male from Lakang Town in early August (Fig. 6A).

**Table 4.** Morphological measurements (in mm) of adult specimens of *Scutigerluozhaensis* sp. nov.

Characters	Holotype CIB 119115	All males (*n* = 13)				All females (*n* = 8)			
		Min	Max	Average	SD	Min	Max	Average	SD
SVL	56.4	47.0	67.2	55.9	4.9	49.8	66.2	59.4	5.5
AG	21.3	17.6	31.7	24.1	3.9	21.4	29.8	27.0	3.5
HL	16.6	15.4	18.6	16.7	0.9	14.4	18.5	17.0	1.5
HW	17.2	16.9	23.1	18.8	1.6	16.4	23.3	20.0	2.6
HH	9.4	6.1	10.3	8.8	1.2	7.3	9.7	8.4	1.0
SL	6.5	6.2	7.9	6.9	0.5	5.8	8.2	7.2	1.0
IND	5.0	4.1	5.7	5.1	0.5	3.8	6.3	5.2	0.8
IOS	4.5	4.1	6.2	4.9	0.5	4.4	7.1	5.2	0.9
UEW	3.8	3.2	5.1	4.0	0.6	3.3	5.3	4.3	0.8
ACED	8.0	6.9	10.5	8.6	0.9	7.0	10.8	9.3	1.4
PCED	13.7	12.5	16.3	14.4	1.1	11.9	17.4	15.4	2.1
ED	5.9	4.4	6.3	5.2	0.6	3.3	6.5	5.4	1.1
SND	3.3	2.6	4.1	3.3	0.5	2.0	4.4	3.3	0.8
END	4.1	2.9	4.3	3.7	0.5	3.3	4.6	4.0	0.5
LAL	14.6	14.1	17.5	15.5	1.2	13.0	16.2	14.8	1.3
LAW	5.6	4.9	6.3	5.6	0.5	3.8	6.6	4.7	0.9
HAL	12.6	12.5	15.1	13.8	1.0	12.9	16.8	14.8	1.3
HLL	78.5	73.3	89.7	80.4	4.5	68.5	84.2	78.0	5.1
THL	21.4	19.7	26.6	22.9	1.8	19.9	25.2	22.3	1.8
TL	21.0	19.6	23.6	21.2	1.2	17.0	21.6	19.8	1.5
TFL	36.0	33.6	39.5	36.3	2.0	31.7	38.4	36.0	2.4
FL	25.0	22.7	27.5	25.4	1.5	22.1	27.9	25.3	2.0
TW	5.9	5.2	6.9	6.0	0.5	4.8	6.4	5.6	0.6
IMTL	4.1	2.4	4.2	3.6	0.7	3.3	5.3	4.1	0.9

##### Sexual dimorphism.

Males are averagely smaller than females, have relatively longer limbs and wider lower arms (Table 4). In adult males, a pair of pectoral glands and a pair of slightly larger axillary glands present on chest, all of them covered by tiny dense black spines in breeding season (Fig. 7). Dorsal surface of first and second fingers, and inner surface of third finger with tiny dense black nuptial spines on adult males in breeding season. Females with a pair of axillary glands, but no spines covered. No observable lineae masculinae present from ventral view of body in males. Tubercles around cloaca on females (e.g., Fig. 6D) fewer and less distinct compared with those on males. Vocal sac and opening absent in both gender.

**Tadpoles.** Their description is based on two tadpoles preserved in 75% ethanol at stage 32 (CIB 119630-1) and 29 (CIB 119630-2) from Lakang Town, Luozha County (Fig. 8, Table 5). Identification of the tadpoles was confirmed by DNA analyses. TOL 40.9–50.7 mm, BL 17.7–18.6 mm, tails length average 152% of body; body elliptical in dorsal view, snout rounded; eyes moderate, dorso-laterally positioned; nostril oval, located in the middle of tip of snout and eyes; oral disc ventrally located; papillae on lips well developed, larger on upper labium; tooth row formula I:3+3/3+3:I or I:3+3/2+2:I; spiraculum sinistral, extended as a short tube, spiracular opening oval; tail musculature robust and greatly narrowing to tail tip. Coloration in preservative greyish brown on dorsal view; semitransparent pale grey on ventral view; tail uniformly pale brown without distinct dark spots.

**Table 5.** Measurements (in mm) of tadpoles of *Scutigerluozhaensis* sp. nov.

Characters	TOL	BL	BH	BW	SNL	SSD	ODW	IOS	TMW	TAL	TMH	HLL
CIB 119630-1	50.70	18.60	5.10	7.80	6.00	9.90	4.50	4.60	3.10	32.10	5.90	2.40
CIB 119630-2	40.90	17.70	4.50	7.00	5.30	8.50	3.90	3.60	3.00	23.30	5.50	1.10
**Characters**	**Stage**	**LTRF**	**BW/BH**	**SSD/BL**	**TAL/BL**	**TMW/BH**	**TMW/TMH**	**TMW/BW**	**ODW/BL**	**ODW/BW**		
CIB 119630-1	32	I: 2+2/3+3: I	1.53	0.53	1.73	1.16	1.90	0.40	0.24	0.58		
CIB 119630-2	29	I: 3+3/3+3: I	1.56	0.48	1.32	1.22	1.83	0.43	0.22	0.56		

##### Distribution and ecology.

**Figure 9.**
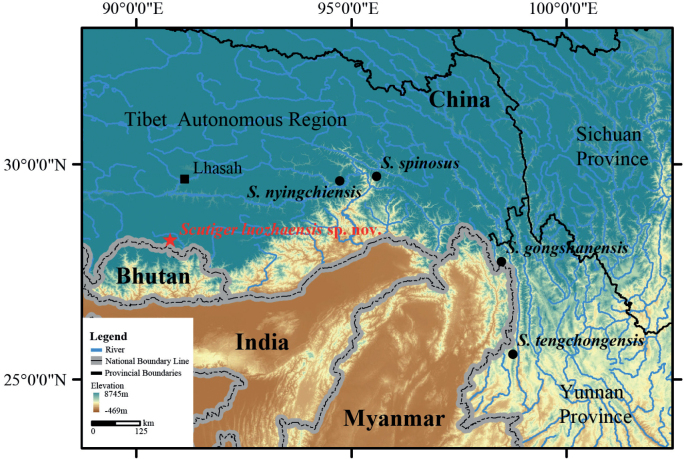
Type localities of *Scutigerluozhaensis* sp. nov. and other species in Clade D.

**Figure 10.**
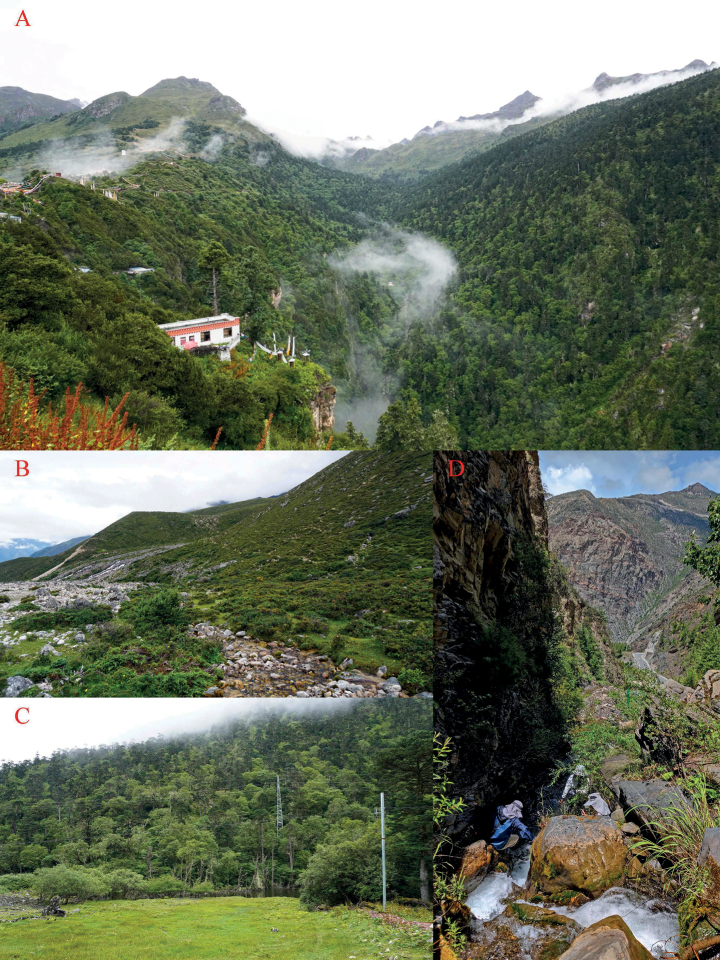
Habitats of *Scutigerluozhaensis* sp. nov. in Luozha County, Tibet, China **A** Qisehai valley in Lakang Town **B** alpine wetlands in Gari Village, Se Town at elevation 4437 m **C** moist mixed coniferous and broad-leaved in Lajiao Town at elevation 3700 m **D** Pugong stream and the Luozha Canyon in Lakang Town at elevation 3268 m. Photographed by SCS.

*Scutigerluozhaensis* sp. nov. is currently only known from Luozha county, Shannan Prefecture, Tibet Autonomous Region, China and expected to be found in adjacent areas of Bhutan (Fig. 9). It is a common species in its habitat, which includes mountain streams, moist scrub or forest floors near streams, and ponds of alpine wetlands (Fig. 10). The elevation records of the new species range from 3268 m to 4437 m. Tadpoles at stages 29, 39, and 48 were recorded in a slack head stream from late July to middle August and it is thought that the tadpoles overwinter. No calls were heard in the field from late July to middle August. Spines on nuptial pads and chest of most adult males had faded when found during field work; the breeding season for the species is here recorded to include June. *Nanoranaparkeri* (Stejneger, 1927) was found to occur with the new species. Although *S.boulengeri* was also found in Luozha County, it was found near an alpine lake at elevation 4623 m, not sympatrically with *S.luozhaensis* sp. nov., which was found restricted to lower elevation.

##### Etymology.

The specific epithet *luozhaensis* is named after the type locality, Luozha county. We propose the English common name Luozha lazy toad and the Chinese name common name 洛扎齿突蟾 (Luò Zhā chǐ Tū Chán).

### ﻿Additional specimens examined in this study

*Scutigerboulengeri* (three adult males): CIB GGS-MGC4-1, CIB GGS-MGC4-9, CIB GGS-MGC4-12 from Kangding, Sichuan, China.

*S.ghunsa* (two adults): male Holotype NHM-TU-17A-0116 and female paratype NHM-TU-17A-0117 from Ghunsa, Taplejung, Nepal.

*S.glandulatus* (three adult males): CIB GGS-PBX2-14, CIB GGS-PBX3-1, CIB GGS-GGSX2-1 from Kangding, Sichuan, China.

*S.gongshanensis* (two adult males): KIZ036221 from Lushui, Nujiuang, Yunnan, China; topotype KIZ036222 from Gongshan, Yunnan, China.

*S.mammatus*, (four adult males from near type locality): CIB GGS-SDX1-1, CIB GGS-PBX4-3, CIB GGS-PBX4-4, GGS-PBX4-5 from Kangding, Sichuan, China.

*S.muliensis* (one adult male): topotypic adult male CIB ML20200727-42 from Muli, Sichuan, China.

*S.nyingchiensis* (ten adults): eight males CIB QZ207, CIB QZ398, CIB QZ401, CIB QZ402, CIB QZ403, CIB QZ408, CIB QZ409, CIB QZ410; two females CIB QZ411, CIB QZ407 from Lulang Town, Bayi District, Tibet, China.

*S.wuguanfui* (six adults of type series): five adult males KIZ030101 (holotype), KIZ030103, KIZ030105, KIZ030106, KIZ030104, adult female KIZ030102 from Medog, Tibet, China.

## ﻿Discussion

### ﻿Cryptic diversity and puzzles in the genus *Scutiger*

*Scutiger* species are distributed in high-altitude regions, such as the Tibetan Plateau, the Himalayas, the Tsingling Mountains, and the Hengduan Mountains. The discovery of *Scutigerluozhaensis* sp. nov. provides additional evidence to support the Paleo-Tibetan origin hypothesis by Hofmann et al. (2017). The unique geomorphic features of the Qinghai-Tibetan Plateau, including rapid uplift and mountainous barrier (Ding et al. 2020; Li et al. 2021; Xu et al. 2021; Miao et al. 2022), may have led to high cryptic species diversity of *Scutiger* in the region, multiple species were not discovered until about recent ten years (Jiang et al. 2012; Jiang et al. 2016; Yang and Huang 2019; Rao "2020", 2022). These specific historical processes and genetic patterns have likely contributed to the diverse and intriguing species patterns observed in *Scutiger* (Chen et al. 2009; Che et al. 2020; Lin et al. 2023), necessitating further research. Due to the limited sampling and genetic data, more research is recommended to fully understand the evolutionary history of *Scutiger*. Such as, there are still puzzles about the *S.boulengeri* species complex, which contained three lineages in this research and as Lin et al. (2023) reported. This also raises the problem of the relationship between *S.bangdaensis* and *S.boulengeri*. The former was described based on morphological data of few specimens only, the diagnostic characters were sorely based on morphological comparisons with *S.boulengeri* and *S.maculatus* without mention of the localities of specimens of *S.boulengeri* compared (Rao "2020", 2022). However, the samples of *S.boulengeri* from Basu (type locality of *S.bangdaensis*) form part of the W.a clade of Lin et al. (2023) with samples from Zaduo (Upper Yangtze Kiang River, near or at the type locality of *S.boulengeri*, and near Chindu, the locality of neotype *S.boulengeri*) and all other samples from Tibet Autonomous Region (Bedriaga 1898; Fei et al. 2009; Lin et al. 2023). One of the morphological diagnoses “absence of spines on ventral belly” was based on specimens collected in October 2016, those spines might have faded in October after breeding season. Other diagnoses “body length 45–50 mm; head width almost equal head length; large tubercles present on dorsolateral body, light colored, and rounded” could not distinctly differ from those specimens of *S.boulengeri* from Tibet Autonomous Region (Fei et al. 2009; Che et al. 2020; Rao 2022 “2020”). These imply that *S.bangdaensis* is possibly a junior synonym of *S.boulengeri*. Further research based on more specimens and genetical data is recommended to solve the puzzles of *S.boulengeri* species complex.

### ﻿The invalidity of *Scutigerbrevipes* (Liu, 1950)

This species was described based on specimens from Taining Town, Daofu County, Sichuan Province, China (Liu 1950). It was synonymized with *S.glandulatus* (Liu, 1950) by Liu and Hu (1961) for the reason that adults of the former in preservative are difficult to differentiate from those of the latter. However, the name *S.glandulatus* was adopted by some researchers probably because the pages describing *S.brevipes* are anterior to those pages describing *S.glandulatus* (Liu 1950; Ye et al. 1992; Fu et al. 1997; Jiang et al. 2012, 2016; Khatiwada et al. 2019; Yang and Huang 2019; Frost 2023). Fei et al. (2009) discussed that *S.brevipes* should remain as a junior synonym of *S.glandulatus*, because "When the precedence between names or nomenclatural acts cannot be objectively determined, the precedence is fixed by the action of the first author citing in a published work those names or acts and selecting from them..." according to article 24.2.1. of the International Code of Zoological Nomenclature (ICZN 1999). Frost (2023) regards *S.brevipes* as a valid species because Fei et al. (2009) did not address the evidence of Fu et al. (1997). However, Fu et al. (1997) re-analyzed the morphological character data of Ye et al. (1992) but never mentioned the name *S.glandulatus*. Other research treated both *S.glandulatus* and *S.brevipes* as valid species but did not provide evidence supporting both names (Jiang et al. 2012, 2016; Khatiwada et al. 2019; Yang and Huang 2019). Thus, there is no evidence supporting the validity of *S.brevipes*, and *S.brevipes* should be retained as a junior synonym of *S.glandulatus*.

### ﻿Conservation implications

Although *Scutigerluozhaensis* is a common species in its habitat, its distribution range may be limited to a small area, including the canyon of Luozha County and possibly adjacent Bhutan. The population size and distribution area for the species are still not clear. The conservation status for this species is suggested to be Data Deficient (DD) and further investigations on this species are recommended.

## Supplementary Material

XML Treatment for Scutiger luozhaensis

## ﻿Additional information

### Author ORCIDs

Sheng-Chao Shi https://orcid.org/0000-0003-2337-6572

Lu-Lu Sui https://orcid.org/0009-0007-5229-5401

Shun Ma https://orcid.org/0009-0003-8611-4550

Fei-Rong Ji https://orcid.org/0009-0005-6900-0556

A-Yi Bu-Dian https://orcid.org/0009-0004-9375-5724

Jian-Ping Jiang https://orcid.org/0000-0002-1051-7797

### Data availability

All of the data that support the findings of this study are available in the main text or Supplementary Information.

## Supplementary materials

Supplementary material 1 Pairwise genetic divergence between lineages of *Scutiger* based on 16S rRNA gene Data type xlsx https://binary.pensoft.net/file/955420This dataset is made available under the Open Database License (http://opendatacommons.org/licenses/odbl/1.0/). The Open Database License (ODbL) is a license agreement intended to allow users to freely share, modify, and use this Dataset while maintaining this same freedom for others, provided that the original source and author(s) are credited.Sheng-Chao Shi, Lu-Lu Sui, Shun Ma, Fei-Rong Ji, A-Yi Bu-Dian, Jian-Ping Jiang

Supplementary material 2 Pairwise genetic divergence between lineages of *Scutiger* based on COI gene Data type xlsx https://binary.pensoft.net/file/955421This dataset is made available under the Open Database License (http://opendatacommons.org/licenses/odbl/1.0/). The Open Database License (ODbL) is a license agreement intended to allow users to freely share, modify, and use this Dataset while maintaining this same freedom for others, provided that the original source and author(s) are credited.Sheng-Chao Shi, Lu-Lu Sui, Shun Ma, Fei-Rong Ji, A-Yi Bu-Dian, Jian-Ping Jiang

Supplementary material 3 Pairwise genetic divergence between lineages of *Scutiger* based on RAG1 gene Data type xlsx https://binary.pensoft.net/file/955422This dataset is made available under the Open Database License (http://opendatacommons.org/licenses/odbl/1.0/). The Open Database License (ODbL) is a license agreement intended to allow users to freely share, modify, and use this Dataset while maintaining this same freedom for others, provided that the original source and author(s) are credited.Sheng-Chao Shi, Lu-Lu Sui, Shun Ma, Fei-Rong Ji, A-Yi Bu-Dian, Jian-Ping Jiang

Supplementary material 4 Morphometrics used for one-way ANOVA analysis Data type xlsx https://binary.pensoft.net/file/955423This dataset is made available under the Open Database License (http://opendatacommons.org/licenses/odbl/1.0/). The Open Database License (ODbL) is a license agreement intended to allow users to freely share, modify, and use this Dataset while maintaining this same freedom for others, provided that the original source and author(s) are credited.Sheng-Chao Shi, Lu-Lu Sui, Shun Ma, Fei-Rong Ji, A-Yi Bu-Dian, Jian-Ping Jiang

Supplementary material 5 Detailed measurements (in mm) for adults of *Scutigerluozhaensis* sp. nov. Data type xlsx https://binary.pensoft.net/file/955424This dataset is made available under the Open Database License (http://opendatacommons.org/licenses/odbl/1.0/). The Open Database License (ODbL) is a license agreement intended to allow users to freely share, modify, and use this Dataset while maintaining this same freedom for others, provided that the original source and author(s) are credited.Sheng-Chao Shi, Lu-Lu Sui, Shun Ma, Fei-Rong Ji, A-Yi Bu-Dian, Jian-Ping Jiang
